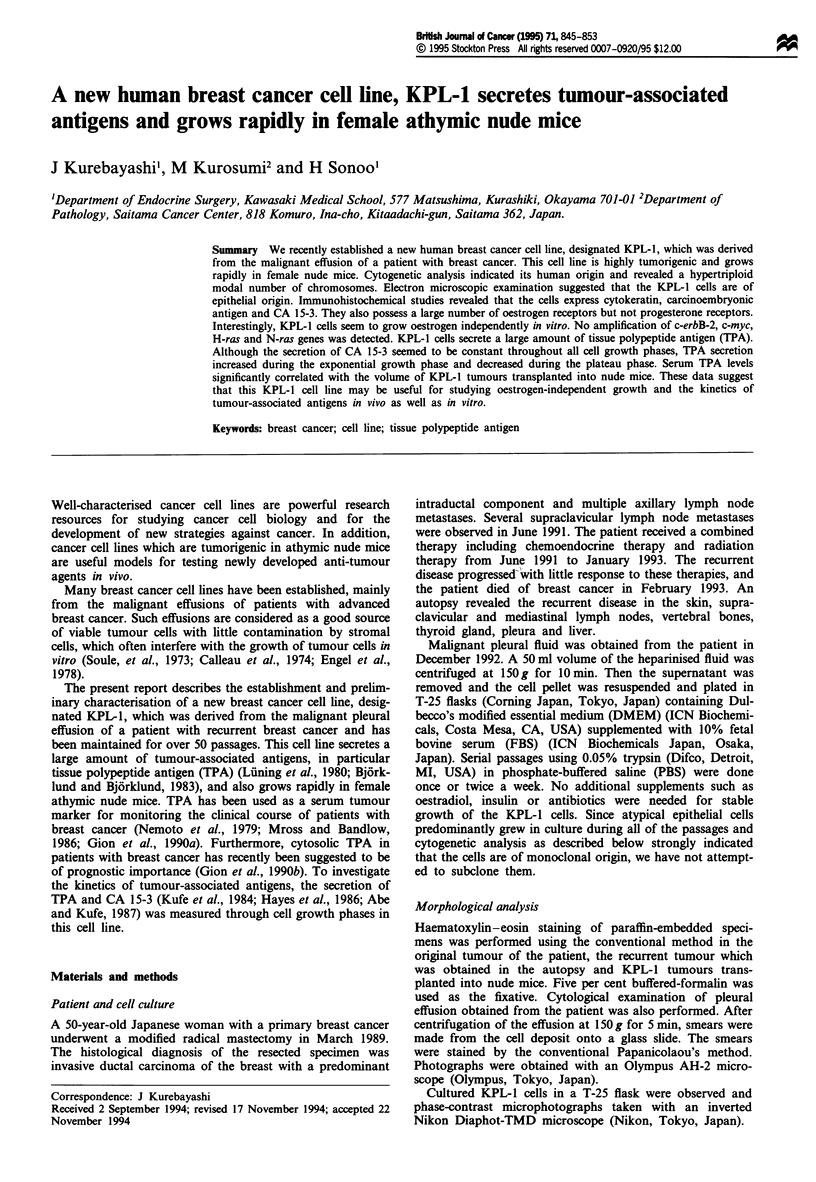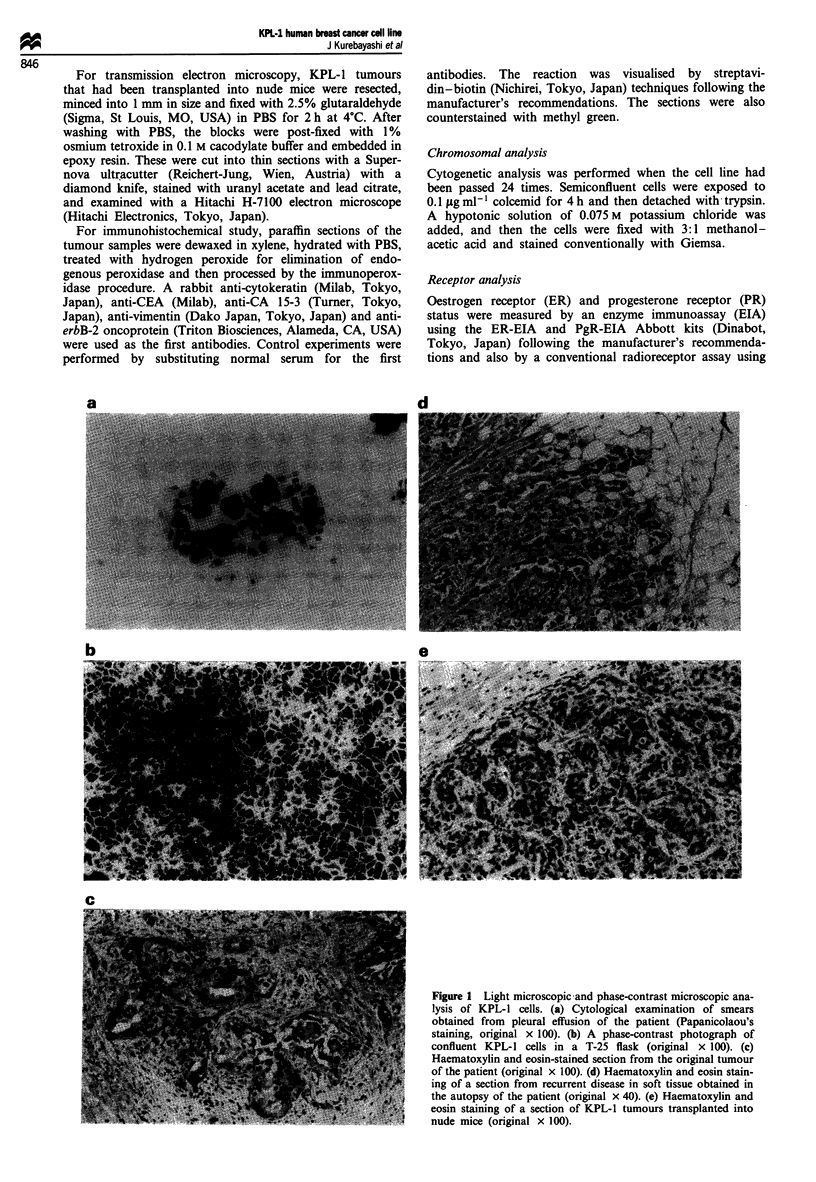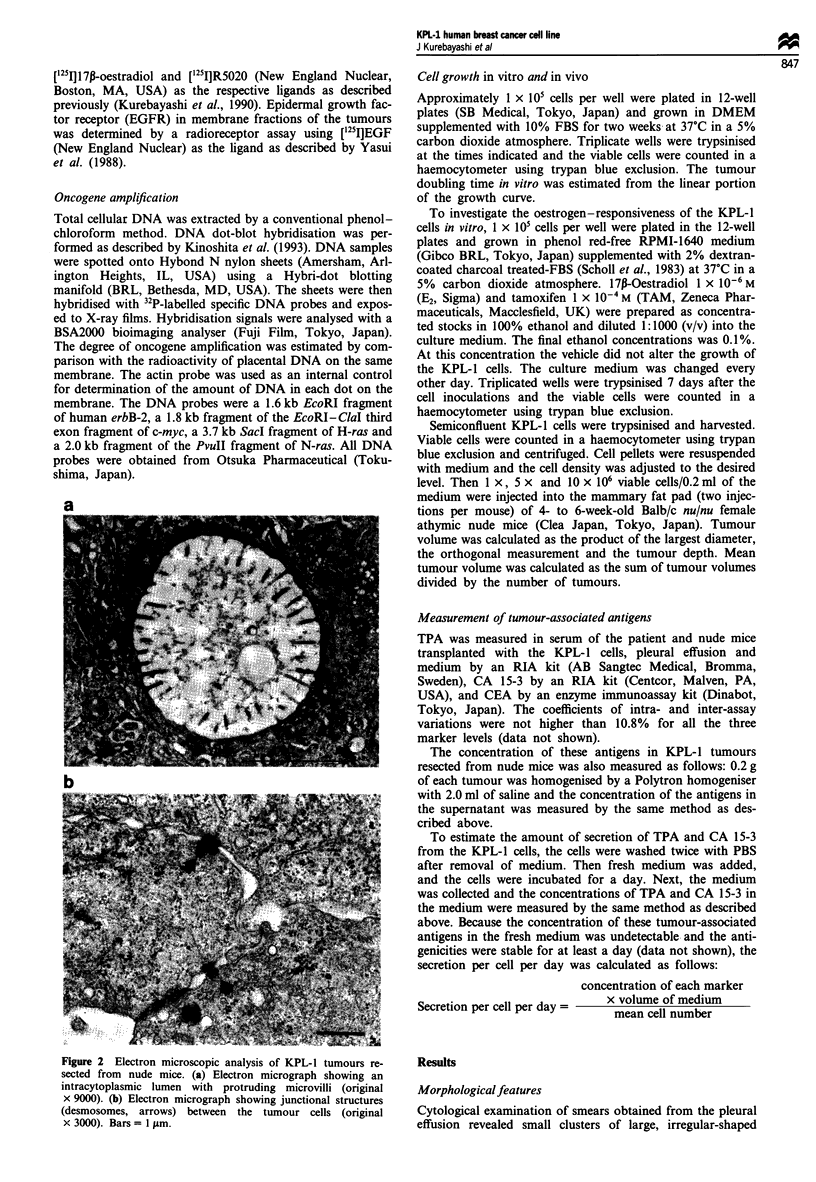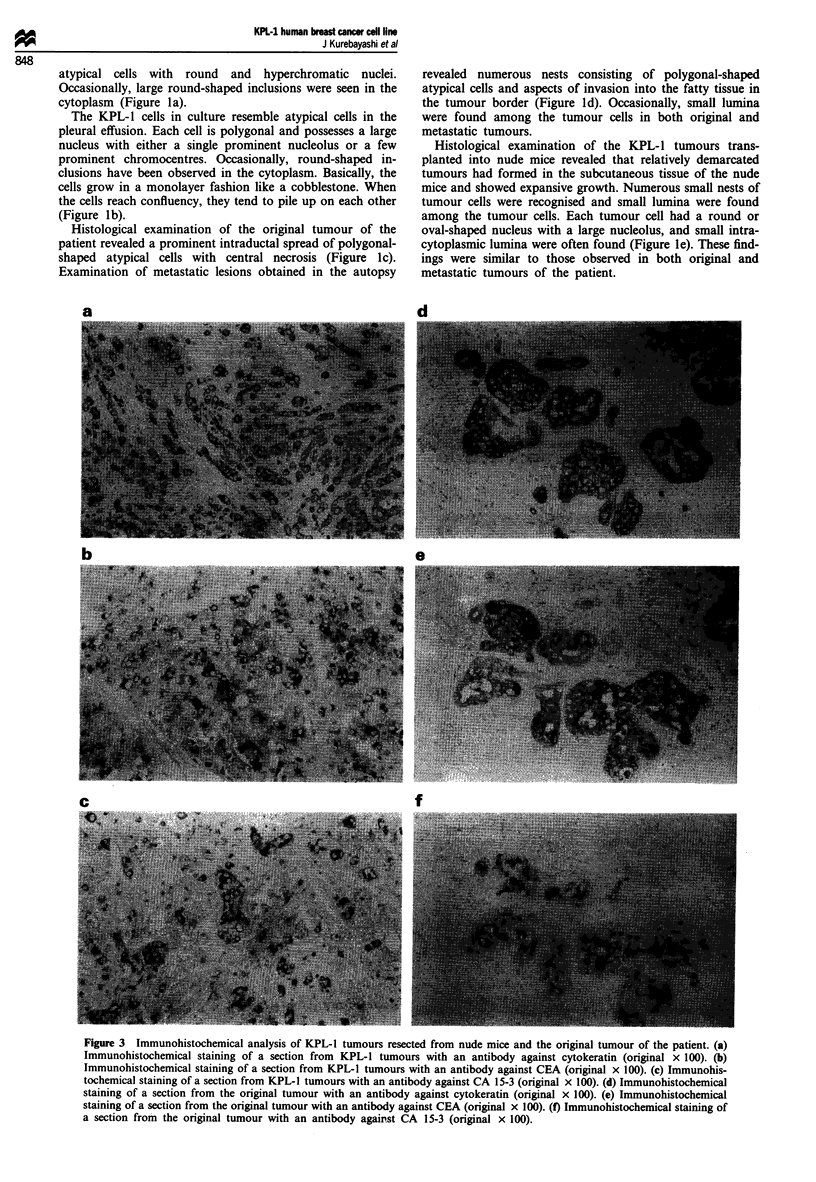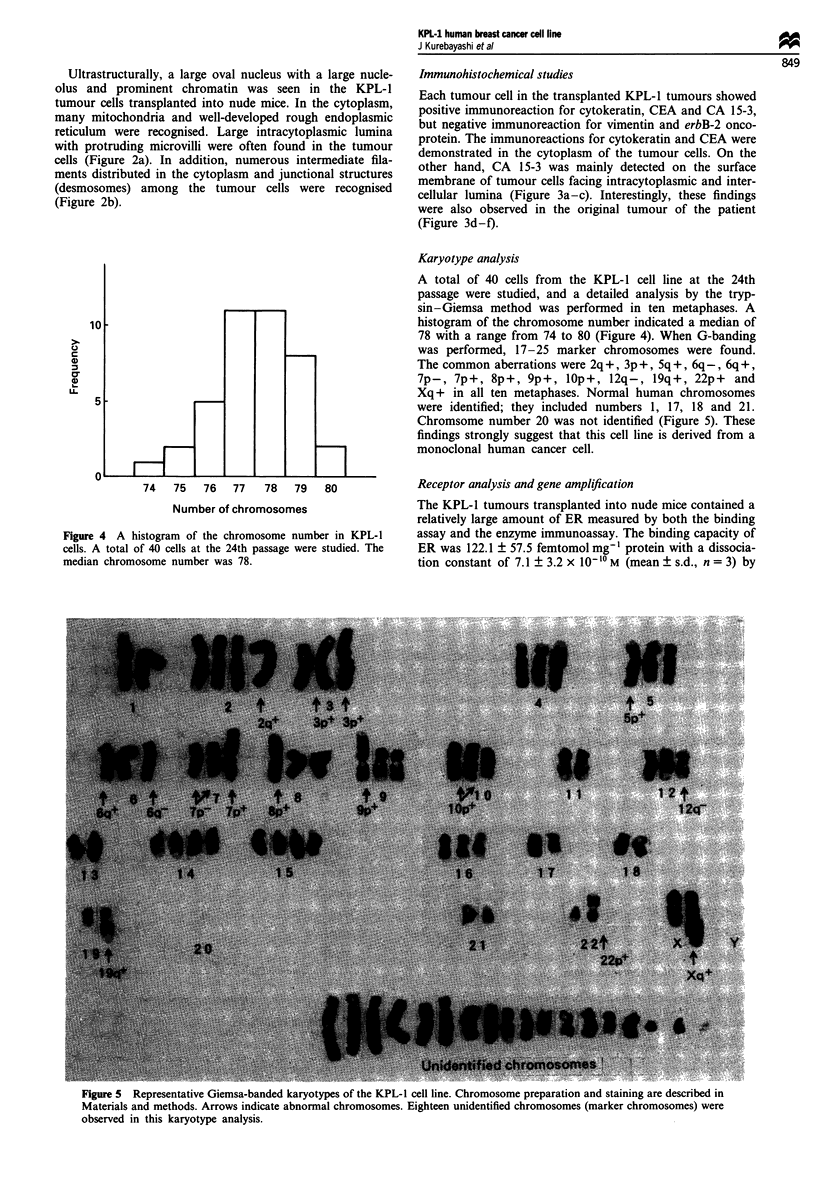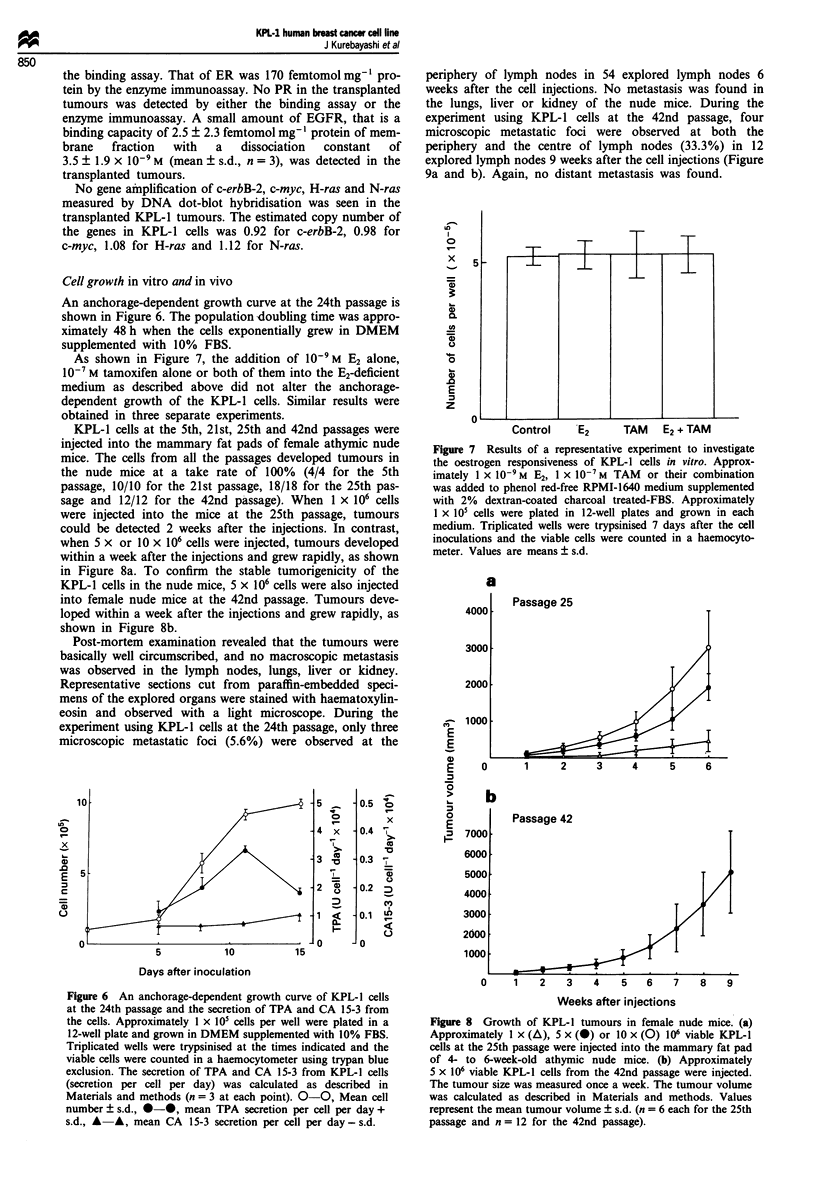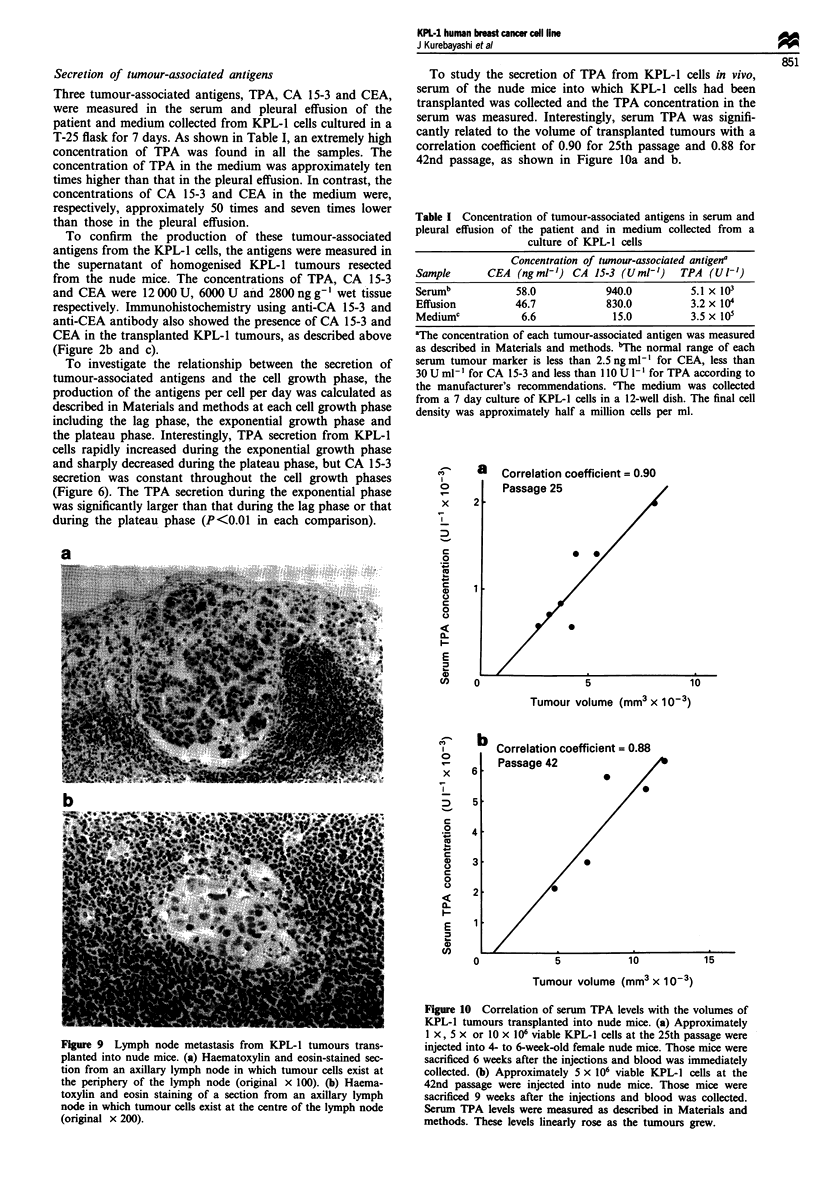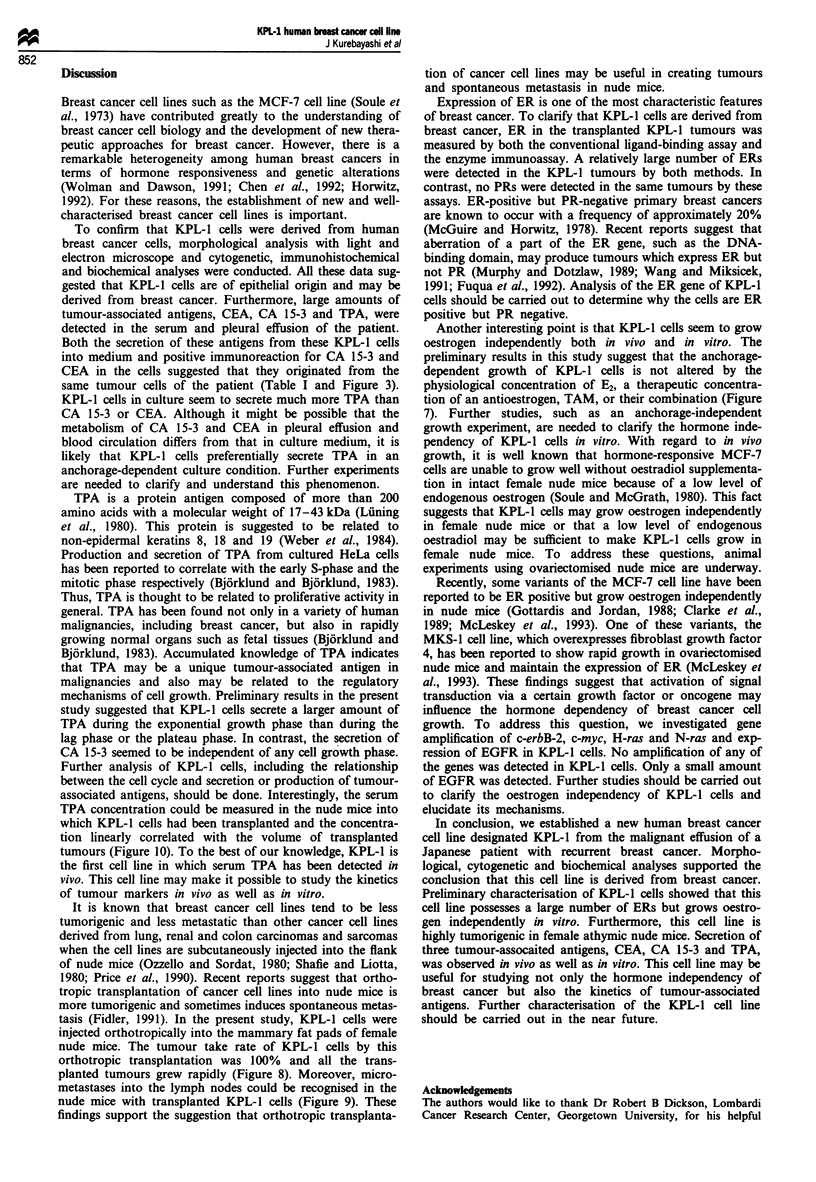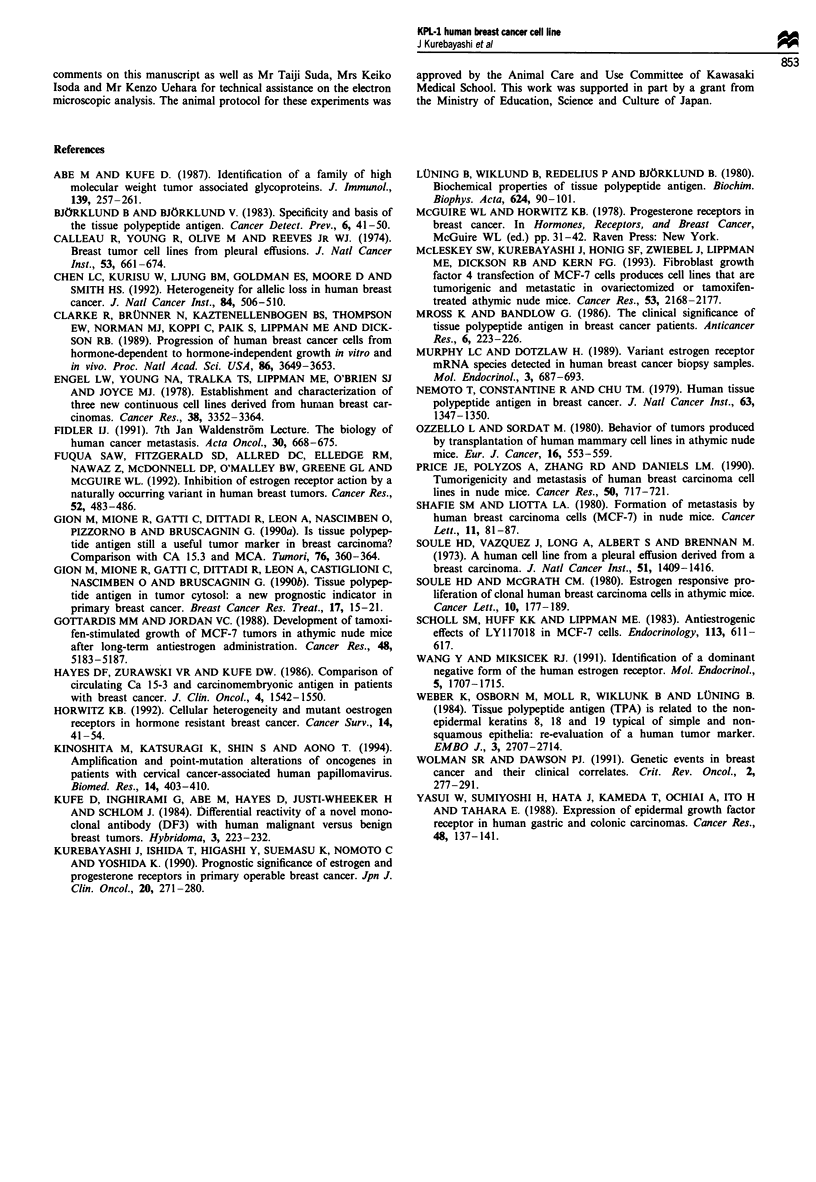# A new human breast cancer cell line, KPL-1 secretes tumour-associated antigens and grows rapidly in female athymic nude mice.

**DOI:** 10.1038/bjc.1995.163

**Published:** 1995-04

**Authors:** J. Kurebayashi, M. Kurosumi, H. Sonoo

**Affiliations:** Department of Endocrine Surgery, Kawasaki Medical School, Okayama, Japan.

## Abstract

**Images:**


British Journal of Cancer (1995) 71, 845-853
© 1995 Stockton Press All rights reserved 0007-0920/95 $12.00

### A new human breast cancer cell line, KPL-1 secretes tumour-associated antigens and grows rapidly in female athymic nude mice

J Kurebayashi[1], M Kurosumi[2] and H Sonoo[1]

[1]*Department of Endocrine Surgery, Kawasaki Medical School, 577 Matsushima, Kurashiki, Okayama 701-01* [2]*Department of Pathology, Saitama Cancer Center, 818 Komuro, Ina-cho, Kitaadachi-gun, Saitama 362, Japan.*

**Summary** We recently established a new human breast cancer cell line, designated KPL-1, which was derived from the malignant effusion of a patient with breast cancer. This cell line is highly tumorigenic and grows rapidly in female nude mice. Cytogenetic analysis indicated its human origin and revealed a hypertriploid modal number of chromosomes. Electron microscopic examination suggested that the KPL-1 cells are of epithelial origin. Immunohistochemical studies revealed that the cells express cytokeratin, carcinoembryonic antigen and CA 15-3. They also possess a large number of oestrogen receptors but not progesterone receptors. Interestingly, KPL-1 cells seem to grow oestrogen independently *in vitro*. No amplification of c-*erb*B-2, c-*myc*, H-*ras* and N-*ras* genes was detected. KPL-1 cells secrete a large amount of tissue polypeptide antigen (TPA). Although the secretion of CA 15-3 seemed to be constant throughout all cell growth phases, TPA secretion increased during the exponential growth phase and decreased during the plateau phase. Serum TPA levels significantly correlated with the volume of KPL-1 tumours transplanted into nude mice. These data suggest that this KPL-1 cell line may be useful for studying oestrogen-independent growth and the kinetics of tumour-associated antigens *in vivo* as well as *in vitro*.

**Keywords:** breast cancer; cell line; tissue polypeptide antigen

Well-characterised cancer cell lines are powerful research resources for studying cancer cell biology and for the development of new strategies against cancer. In addition, cancer cell lines which are tumorigenic in athymic nude mice are useful models for testing newly developed anti-tumour agents *in vivo*.

Many breast cancer cell lines have been established, mainly from the malignant effusions of patients with advanced breast cancer. Such effusions are considered as a good source of viable tumour cells with little contamination by stromal cells, which often interfere with the growth of tumour cells *in vitro* (Soule, *et al.*, 1973; Calleau *et al.*, 1974; Engel *et al.*, 1978).

The present report describes the establishment and preliminary characterisation of a new breast cancer cell line, designated KPL-1, which was derived from the malignant pleural effusion of a patient with recurrent breast cancer and has been maintained for over 50 passages. This cell line secretes a large amount of tumour-associated antigens, in particular tissue polypeptide antigen (TPA) (Lüning *et al.*, 1980; Björklund and Björklund, 1983), and also grows rapidly in female athymic nude mice. TPA has been used as a serum tumour marker for monitoring the clinical course of patients with breast cancer (Nemoto *et al.*, 1979; Mross and Bandlow, 1986; Gion *et al.*, 1990*a*). Furthermore, cytosolic TPA in patients with breast cancer has recently been suggested to be of prognostic importance (Gion *et al.*, 1990*b*). To investigate the kinetics of tumour-associated antigens, the secretion of TPA and CA 15-3 (Kufe *et al.*, 1984; Hayes *et al.*, 1986; Abe and Kufe, 1987) was measured through cell growth phases in this cell line.

### Materials and methods

### Patient and cell culture

A 50-year-old Japanese woman with a primary breast cancer underwent a modified radical mastectomy in March 1989. The histological diagnosis of the resected specimen was invasive ductal carcinoma of the breast with a predominant intraductal component and multiple axillary lymph node metastases. Several supraclavicular lymph node metastases were observed in June 1991. The patient received a combined therapy including chemoendocrine therapy and radiation therapy from June 1991 to January 1993. The recurrent disease progressed with little response to these therapies, and the patient died of breast cancer in February 1993. An autopsy revealed the recurrent disease in the skin, supraclavicular and mediastinal lymph nodes, vertebral bones, thyroid gland, pleura and liver.

Malignant pleural fluid was obtained from the patient in December 1992. A 50 ml volume of the heparinised fluid was centrifuged at 150 *g* for 10 min. Then the supernatant was removed and the cell pellet was resuspended and plated in T-25 flasks (Corning Japan, Tokyo, Japan) containing Dulbecco's modified essential medium (DMEM) (ICN Biochemicals, Costa Mesa, CA, USA) supplemented with 10% fetal bovine serum (FBS) (ICN Biochemicals Japan, Osaka, Japan). Serial passages using 0.05% trypsin (Difco, Detroit, MI, USA) in phosphate-buffered saline (PBS) were done once or twice a week. No additional supplements such as oestradiol, insulin or antibiotics were needed for stable growth of the KPL-1 cells. Since atypical epithelial cells predominantly grew in culture during all of the passages and cytogenetic analysis as described below strongly indicated that the cells are of monoclonal origin, we have not attempted to subclone them.

### Morphological analysis

Haematoxylin–eosin staining of paraffin-embedded specimens was performed using the conventional method in the original tumour of the patient, the recurrent tumour which was obtained in the autopsy and KPL-1 tumours transplanted into nude mice. Five per cent buffered-formalin was used as the fixative. Cytological examination of pleural effusion obtained from the patient was also performed. After centrifugation of the effusion at 150 *g* for 5 min, smears were made from the cell deposit onto a glass slide. The smears were stained by the conventional Papanicolaou's method. Photographs were obtained with an Olympus AH-2 microscope (Olympus, Tokyo, Japan).

Cultured KPL-1 cells in a T-25 flask were observed and phase-contrast microphotographs taken with an inverted Nikon Diaphot-TMD microscope (Nikon, Tokyo, Japan).

Correspondence: J Kurebayashi
Received 2 September 1994; revised 17 November 1994; accepted 22 November 1994

For transmission electron microscopy, KPL-1 tumours that had been transplanted into nude mice were resected, minced into 1 mm in size and fixed with 2.5% glutaraldehyde (Sigma, St Louis, MO, USA) in PBS for 2 h at 4°C. After washing with PBS, the blocks were post-fixed with 1% osmium tetroxide in 0.1 M cacodylate buffer and embedded in epoxy resin. These were cut into thin sections with a Supernova ultracutter (Reichert-Jung, Wien, Austria) with a diamond knife, stained with uranyl acetate and lead citrate, and examined with a Hitachi H-7100 electron microscope (Hitachi Electronics, Tokyo, Japan).

For immunohistochemical study, paraffin sections of the tumour samples were dewaxed in xylene, hydrated with PBS, treated with hydrogen peroxide for elimination of endogenous peroxidase and then processed by the immunoperoxidase procedure. A rabbit anti-cytokeratin (Milab, Tokyo, Japan), anti-CEA (Milab), anti-CA 15-3 (Turner, Tokyo, Japan), anti-vimentin (Dako Japan, Tokyo, Japan) and anti-*erb*B-2 oncoprotein (Triton Biosciences, Alameda, CA, USA) were used as the first antibodies. Control experiments were performed by substituting normal serum for the first antibodies. The reaction was visualised by streptavidin–biotin (Nichirei, Tokyo, Japan) techniques following the manufacturer's recommendations. The sections were also counterstained with methyl green.

### *Chromosomal analysis*

Cytogenetic analysis was performed when the cell line had been passed 24 times. Semiconfluent cells were exposed to 0.1 $\mu g\ ml^{-1}$ colcemid for 4 h and then detached with trypsin. A hypotonic solution of 0.075 M potassium chloride was added, and then the cells were fixed with 3:1 methanol–acetic acid and stained conventionally with Giemsa.

### *Receptor analysis*

Oestrogen receptor (ER) and progesterone receptor (PR) status were measured by an enzyme immunoassay (EIA) using the ER-EIA and PgR-EIA Abbott kits (Dinabot, Tokyo, Japan) following the manufacturer's recommendations and also by a conventional radioreceptor assay using

**Figure 1** Light microscopic and phase-contrast microscopic analysis of KPL-1 cells. (**a**) Cytological examination of smears obtained from pleural effusion of the patient (Papanicolaou's staining, original × 100). (**b**) A phase-contrast photograph of confluent KPL-1 cells in a T-25 flask (original × 100). (**c**) Haematoxylin and eosin-stained section from the original tumour of the patient (original × 100). (**d**) Haematoxylin and eosin staining of a section from recurrent disease in soft tissue obtained in the autopsy of the patient (original × 40). (**e**) Haematoxylin and eosin staining of a section of KPL-1 tumours transplanted into nude mice (original × 100).

[$^{125}$I]17β-oestradiol and [$^{125}$I]R5020 (New England Nuclear, Boston, MA, USA) as the respective ligands as described previously (Kurebayashi *et al.*, 1990). Epidermal growth factor receptor (EGFR) in membrane fractions of the tumours was determined by a radioreceptor assay using [$^{125}$I]EGF (New England Nuclear) as the ligand as described by Yasui *et al.* (1988).

*Oncogene amplification*

Total cellular DNA was extracted by a conventional phenol–chloroform method. DNA dot-blot hybridisation was performed as described by Kinoshita *et al.* (1993). DNA samples were spotted onto Hybond N nylon sheets (Amersham, Arlington Heights, IL, USA) using a Hybri-dot blotting manifold (BRL, Bethesda, MD, USA). The sheets were then hybridised with $^{32}$P-labelled specific DNA probes and exposed to X-ray films. Hybridisation signals were analysed with a BSA2000 bioimaging analyser (Fuji Film, Tokyo, Japan). The degree of oncogene amplification was estimated by comparison with the radioactivity of placental DNA on the same membrane. The actin probe was used as an internal control for determination of the amount of DNA in each dot on the membrane. The DNA probes were a 1.6 kb *Eco*RI fragment of human *erb*B-2, a 1.8 kb fragment of the *Eco*RI–*Cla*I third exon fragment of c-*myc*, a 3.7 kb *Sac*I fragment of H-*ras* and a 2.0 kb fragment of the *Pvu*II fragment of N-*ras*. All DNA probes were obtained from Otsuka Pharmaceutical (Tokushima, Japan).

**Figure 2** Electron microscopic analysis of KPL-1 tumours resected from nude mice. (**a**) Electron micrograph showing an intracytoplasmic lumen with protruding microvilli (original × 9000). (**b**) Electron micrograph showing junctional structures (desmosomes, arrows) between the tumour cells (original × 3000). Bars = 1 μm.

*Cell growth* in vitro *and* in vivo

Approximately $1 \times 10^5$ cells per well were plated in 12-well plates (SB Medical, Tokyo, Japan) and grown in DMEM supplemented with 10% FBS for two weeks at 37°C in a 5% carbon dioxide atmosphere. Triplicate wells were trypsinised at the times indicated and the viable cells were counted in a haemocytometer using trypan blue exclusion. The tumour doubling time *in vitro* was estimated from the linear portion of the growth curve.

To investigate the oestrogen–responsiveness of the KPL-1 cells *in vitro*, $1 \times 10^5$ cells per well were plated in the 12-well plates and grown in phenol red-free RPMI-1640 medium (Gibco BRL, Tokyo, Japan) supplemented with 2% dextran-coated charcoal treated-FBS (Scholl *et al.*, 1983) at 37°C in a 5% carbon dioxide atmosphere. 17β-Oestradiol $1 \times 10^{-6}$ M ($E_2$, Sigma) and tamoxifen $1 \times 10^{-4}$ M (TAM, Zeneca Pharmaceuticals, Macclesfield, UK) were prepared as concentrated stocks in 100% ethanol and diluted 1:1000 (v/v) into the culture medium. The final ethanol concentrations was 0.1%. At this concentration the vehicle did not alter the growth of the KPL-1 cells. The culture medium was changed every other day. Triplicated wells were trypsinised 7 days after the cell inoculations and the viable cells were counted in a haemocytometer using trypan blue exclusion.

Semiconfluent KPL-1 cells were trypsinised and harvested. Viable cells were counted in a haemocytometer using trypan blue exclusion and centrifuged. Cell pellets were resuspended with medium and the cell density was adjusted to the desired level. Then 1 ×, 5 × and $10 \times 10^6$ viable cells/0.2 ml of the medium were injected into the mammary fat pad (two injections per mouse) of 4- to 6-week-old Balb/c *nu/nu* female athymic nude mice (Clea Japan, Tokyo, Japan). Tumour volume was calculated as the product of the largest diameter, the orthogonal measurement and the tumour depth. Mean tumour volume was calculated as the sum of tumour volumes divided by the number of tumours.

*Measurement of tumour-associated antigens*

TPA was measured in serum of the patient and nude mice transplanted with the KPL-1 cells, pleural effusion and medium by an RIA kit (AB Sangtec Medical, Bromma, Sweden), CA 15-3 by an RIA kit (Centcor, Malven, PA, USA), and CEA by an enzyme immunoassay kit (Dinabot, Tokyo, Japan). The coefficients of intra- and inter-assay variations were not higher than 10.8% for all the three marker levels (data not shown).

The concentration of these antigens in KPL-1 tumours resected from nude mice was also measured as follows: 0.2 g of each tumour was homogenised by a Polytron homogeniser with 2.0 ml of saline and the concentration of the antigens in the supernatant was measured by the same method as described above.

To estimate the amount of secretion of TPA and CA 15-3 from the KPL-1 cells, the cells were washed twice with PBS after removal of medium. Then fresh medium was added, and the cells were incubated for a day. Next, the medium was collected and the concentrations of TPA and CA 15-3 in the medium were measured by the same method as described above. Because the concentration of these tumour-associated antigens in the fresh medium was undetectable and the antigenicities were stable for at least a day (data not shown), the secretion per cell per day was calculated as follows:

$$\text{Secretion per cell per day} = \frac{\text{concentration of each marker} \times \text{volume of medium}}{\text{mean cell number}}$$

### Results

*Morphological features*

Cytological examination of smears obtained from the pleural effusion revealed small clusters of large, irregular-shaped atypical cells with round and hyperchromatic nuclei. Occasionally, large round-shaped inclusions were seen in the cytoplasm (Figure 1a).

The KPL-1 cells in culture resemble atypical cells in the pleural effusion. Each cell is polygonal and possesses a large nucleus with either a single prominent nucleolus or a few prominent chromocentres. Occasionally, round-shaped inclusions have been observed in the cytoplasm. Basically, the cells grow in a monolayer fashion like a cobblestone. When the cells reach confluency, they tend to pile up on each other (Figure 1b).

Histological examination of the original tumour of the patient revealed a prominent intraductal spread of polygonal-shaped atypical cells with central necrosis (Figure 1c). Examination of metastatic lesions obtained in the autopsy revealed numerous nests consisting of polygonal-shaped atypical cells and aspects of invasion into the fatty tissue in the tumour border (Figure 1d). Occasionally, small lumina were found among the tumour cells in both original and metastatic tumours.

Histological examination of the KPL-1 tumours transplanted into nude mice revealed that relatively demarcated tumours had formed in the subcutaneous tissue of the nude mice and showed expansive growth. Numerous small nests of tumour cells were recognised and small lumina were found among the tumour cells. Each tumour cell had a round or oval-shaped nucleus with a large nucleolus, and small intracytoplasmic lumina were often found (Figure 1e). These findings were similar to those observed in both original and metastatic tumours of the patient.

**Figure 3** Immunohistochemical analysis of KPL-1 tumours resected from nude mice and the original tumour of the patient. (**a**) Immunohistochemical staining of a section from KPL-1 tumours with an antibody against cytokeratin (original × 100). (**b**) Immunohistochemical staining of a section from KPL-1 tumours with an antibody against CEA (original × 100). (**c**) Immunohistochemical staining of a section from KPL-1 tumours with an antibody against CA 15-3 (original × 100). (**d**) Immunohistochemical staining of a section from the original tumour with an antibody against cytokeratin (original × 100). (**e**) Immunohistochemical staining of a section from the original tumour with an antibody against CEA (original × 100). (**f**) Immunohistochemical staining of a section from the original tumour with an antibody against CA 15-3 (original × 100).

Ultrastructurally, a large oval nucleus with a large nucleolus and prominent chromatin was seen in the KPL-1 tumour cells transplanted into nude mice. In the cytoplasm, many mitochondria and well-developed rough endoplasmic reticulum were recognised. Large intracytoplasmic lumina with protruding microvilli were often found in the tumour cells (Figure 2a). In addition, numerous intermediate filaments distributed in the cytoplasm and junctional structures (desmosomes) among the tumour cells were recognised (Figure 2b).

**Figure 4** A histogram of the chromosome number in KPL-1 cells. A total of 40 cells at the 24th passage were studied. The median chromosome number was 78.

*Immunohistochemical studies*

Each tumour cell in the transplanted KPL-1 tumours showed positive immunoreaction for cytokeratin, CEA and CA 15-3, but negative immunoreaction for vimentin and *erb*B-2 oncoprotein. The immunoreactions for cytokeratin and CEA were demonstrated in the cytoplasm of the tumour cells. On the other hand, CA 15-3 was mainly detected on the surface membrane of tumour cells facing intracytoplasmic and intercellular lumina (Figure 3a–c). Interestingly, these findings were also observed in the original tumour of the patient (Figure 3d–f).

*Karyotype analysis*

A total of 40 cells from the KPL-1 cell line at the 24th passage were studied, and a detailed analysis by the trypsin–Giemsa method was performed in ten metaphases. A histogram of the chromosome number indicated a median of 78 with a range from 74 to 80 (Figure 4). When G-banding was performed, 17–25 marker chromosomes were found. The common aberrations were 2q+, 3p+, 5q+, 6q−, 6q+, 7p−, 7p+, 8p+, 9p+, 10p+, 12q−, 19q+, 22p+ and Xq+ in all ten metaphases. Normal human chromosomes were identified; they included numbers 1, 17, 18 and 21. Chromsome number 20 was not identified (Figure 5). These findings strongly suggest that this cell line is derived from a monoclonal human cancer cell.

*Receptor analysis and gene amplification*

The KPL-1 tumours transplanted into nude mice contained a relatively large amount of ER measured by both the binding assay and the enzyme immunoassay. The binding capacity of ER was 122.1 ± 57.5 femtomol mg$^{-1}$ protein with a dissociation constant of 7.1 ± 3.2 × 10$^{-10}$ M (mean ± s.d., $n = 3$) by

**Figure 5** Representative Giemsa-banded karyotypes of the KPL-1 cell line. Chromosome preparation and staining are described in Materials and methods. Arrows indicate abnormal chromosomes. Eighteen unidentified chromosomes (marker chromosomes) were observed in this karyotype analysis.

the binding assay. That of ER was 170 femtomol $mg^{-1}$ protein by the enzyme immunoassay. No PR in the transplanted tumours was detected by either the binding assay or the enzyme immunoassay. A small amount of EGFR, that is a binding capacity of 2.5 ± 2.3 femtomol $mg^{-1}$ protein of membrane fraction with a dissociation constant of $3.5 \pm 1.9 \times 10^{-9}$ M (mean ± s.d., $n = 3$), was detected in the transplanted tumours.

No gene amplification of c-*erb*B-2, c-*myc*, H-*ras* and N-*ras* measured by DNA dot-blot hybridisation was seen in the transplanted KPL-1 tumours. The estimated copy number of the genes in KPL-1 cells was 0.92 for c-*erb*B-2, 0.98 for c-*myc*, 1.08 for H-*ras* and 1.12 for N-*ras*.

### *Cell growth* in vitro *and* in vivo

An anchorage-dependent growth curve at the 24th passage is shown in Figure 6. The population doubling time was approximately 48 h when the cells exponentially grew in DMEM supplemented with 10% FBS.

As shown in Figure 7, the addition of $10^{-9}$ M $E_2$ alone, $10^{-7}$ M tamoxifen alone or both of them into the $E_2$-deficient medium as described above did not alter the anchorage-dependent growth of the KPL-1 cells. Similar results were obtained in three separate experiments.

KPL-1 cells at the 5th, 21st, 25th and 42nd passages were injected into the mammary fat pads of female athymic nude mice. The cells from all the passages developed tumours in the nude mice at a take rate of 100% (4/4 for the 5th passage, 10/10 for the 21st passage, 18/18 for the 25th passage and 12/12 for the 42nd passage). When $1 \times 10^6$ cells were injected into the mice at the 25th passage, tumours could be detected 2 weeks after the injections. In contrast, when 5 × or 10 × $10^6$ cells were injected, tumours developed within a week after the injections and grew rapidly, as shown in Figure 8a. To confirm the stable tumorigenicity of the KPL-1 cells in the nude mice, $5 \times 10^6$ cells were also injected into female nude mice at the 42nd passage. Tumours developed within a week after the injections and grew rapidly, as shown in Figure 8b.

Post-mortem examination revealed that the tumours were basically well circumscribed, and no macroscopic metastasis was observed in the lymph nodes, lungs, liver or kidney. Representative sections cut from paraffin-embedded specimens of the explored organs were stained with haematoxylin-eosin and observed with a light microscope. During the experiment using KPL-1 cells at the 24th passage, only three microscopic metastatic foci (5.6%) were observed at the periphery of lymph nodes in 54 explored lymph nodes 6 weeks after the cell injections. No metastasis was found in the lungs, liver or kidney of the nude mice. During the experiment using KPL-1 cells at the 42nd passage, four microscopic metastatic foci were observed at both the periphery and the centre of lymph nodes (33.3%) in 12 explored lymph nodes 9 weeks after the cell injections (Figure 9a and b). Again, no distant metastasis was found.

**Figure 6** An anchorage-dependent growth curve of KPL-1 cells at the 24th passage and the secretion of TPA and CA 15-3 from the cells. Approximately $1 \times 10^5$ cells per well were plated in a 12-well plate and grown in DMEM supplemented with 10% FBS. Triplicated wells were trypsinised at the times indicated and the viable cells were counted in a haemocytometer using trypan blue exclusion. The secretion of TPA and CA 15-3 from KPL-1 cells (secretion per cell per day) was calculated as described in Materials and methods ($n = 3$ at each point). O—O, Mean cell number ± s.d., ●—●, mean TPA secretion per cell per day + s.d., ▲—▲, mean CA 15-3 secretion per cell per day − s.d.

**Figure 7** Results of a representative experiment to investigate the oestrogen responsiveness of KPL-1 cells *in vitro*. Approximately $1 \times 10^{-9}$ M $E_2$, $1 \times 10^{-7}$ M TAM or their combination was added to phenol red-free RPMI-1640 medium supplemented with 2% dextran-coated charcoal treated-FBS. Approximately $1 \times 10^5$ cells were plated in 12-well plates and grown in each medium. Triplicated wells were trypsinised 7 days after the cell inoculations and the viable cells were counted in a haemocytometer. Values are means ± s.d.

**Figure 8** Growth of KPL-1 tumours in female nude mice. (**a**) Approximately 1 × (Δ), 5 × (●) or 10 × (O) $10^6$ viable KPL-1 cells at the 25th passage were injected into the mammary fat pad of 4- to 6-week-old athymic nude mice. (**b**) Approximately $5 \times 10^6$ viable KPL-1 cells from the 42nd passage were injected. The tumour size was measured once a week. The tumour volume was calculated as described in Materials and methods. Values represent the mean tumour volume ± s.d. ($n = 6$ each for the 25th passage and $n = 12$ for the 42nd passage).

*Secretion of tumour-associated antigens*

Three tumour-associated antigens, TPA, CA 15-3 and CEA, were measured in the serum and pleural effusion of the patient and medium collected from KPL-1 cells cultured in a T-25 flask for 7 days. As shown in Table I, an extremely high concentration of TPA was found in all the samples. The concentration of TPA in the medium was approximately ten times higher than that in the pleural effusion. In contrast, the concentrations of CA 15-3 and CEA in the medium were, respectively, approximately 50 times and seven times lower than those in the pleural effusion.

To confirm the production of these tumour-associated antigens from the KPL-1 cells, the antigens were measured in the supernatant of homogenised KPL-1 tumours resected from the nude mice. The concentrations of TPA, CA 15-3 and CEA were 12 000 U, 6000 U and 2800 ng $g^{-1}$ wet tissue respectively. Immunohistochemistry using anti-CA 15-3 and anti-CEA antibody also showed the presence of CA 15-3 and CEA in the transplanted KPL-1 tumours, as described above (Figure 2b and c).

To investigate the relationship between the secretion of tumour-associated antigens and the cell growth phase, the production of the antigens per cell per day was calculated as described in Materials and methods at each cell growth phase including the lag phase, the exponential growth phase and the plateau phase. Interestingly, TPA secretion from KPL-1 cells rapidly increased during the exponential growth phase and sharply decreased during the plateau phase, but CA 15-3 secretion was constant throughout the cell growth phases (Figure 6). The TPA secretion during the exponential phase was significantly larger than that during the lag phase or that during the plateau phase ($P<0.01$ in each comparison).

**Figure 9** Lymph node metastasis from KPL-1 tumours transplanted into nude mice. (**a**) Haematoxylin and eosin-stained section from an axillary lymph node in which tumour cells exist at the periphery of the lymph node (original × 100). (**b**) Haematoxylin and eosin staining of a section from an axillary lymph node in which tumour cells exist at the centre of the lymph node (original × 200).

To study the secretion of TPA from KPL-1 cells *in vivo*, serum of the nude mice into which KPL-1 cells had been transplanted was collected and the TPA concentration in the serum was measured. Interestingly, serum TPA was significantly related to the volume of transplanted tumours with a correlation coefficient of 0.90 for 25th passage and 0.88 for 42nd passage, as shown in Figure 10a and b.

**Table I** Concentration of tumour-associated antigens in serum and pleural effusion of the patient and in medium collected from a culture of KPL-1 cells

| | *Concentration of tumour-associated antigen*[a] | | |
|---|---|---|---|
| *Sample* | *CEA (ng $ml^{-1}$)* | *CA 15-3 (U $ml^{-1}$)* | *TPA (U $l^{-1}$)* |
| Serum[b] | 58.0 | 940.0 | $5.1 \times 10^3$ |
| Effusion | 46.7 | 830.0 | $3.2 \times 10^4$ |
| Medium[c] | 6.6 | 15.0 | $3.5 \times 10^5$ |

[a]The concentration of each tumour-associated antigen was measured as described in Materials and methods. [b]The normal range of each serum tumour marker is less than 2.5 ng $ml^{-1}$ for CEA, less than 30 U $ml^{-1}$ for CA 15-3 and less than 110 U $l^{-1}$ for TPA according to the manufacturer's recommendations. [c]The medium was collected from a 7 day culture of KPL-1 cells in a 12-well dish. The final cell density was approximately half a million cells per ml.

**Figure 10** Correlation of serum TPA levels with the volumes of KPL-1 tumours transplanted into nude mice. (**a**) Approximately 1 ×, 5 × or 10 × $10^6$ viable KPL-1 cells at the 25th passage were injected into 4- to 6-week-old female nude mice. Those mice were sacrificed 6 weeks after the injections and blood was immediately collected. (**b**) Approximately 5 × $10^6$ viable KPL-1 cells at the 42nd passage were injected into nude mice. Those mice were sacrificed 9 weeks after the injections and blood was collected. Serum TPA levels were measured as described in Materials and methods. These levels linearly rose as the tumours grew.

### Discussion

Breast cancer cell lines such as the MCF-7 cell line (Soule *et al.*, 1973) have contributed greatly to the understanding of breast cancer cell biology and the development of new therapeutic approaches for breast cancer. However, there is a remarkable heterogeneity among human breast cancers in terms of hormone responsiveness and genetic alterations (Wolman and Dawson, 1991; Chen *et al.*, 1992; Horwitz, 1992). For these reasons, the establishment of new and well-characterised breast cancer cell lines is important.

To confirm that KPL-1 cells were derived from human breast cancer cells, morphological analysis with light and electron microscope and cytogenetic, immunohistochemical and biochemical analyses were conducted. All these data suggested that KPL-1 cells are of epithelial origin and may be derived from breast cancer. Furthermore, large amounts of tumour-associated antigens, CEA, CA 15-3 and TPA, were detected in the serum and pleural effusion of the patient. Both the secretion of these antigens from these KPL-1 cells into medium and positive immunoreaction for CA 15-3 and CEA in the cells suggested that they originated from the same tumour cells of the patient (Table I and Figure 3). KPL-1 cells in culture seem to secrete much more TPA than CA 15-3 or CEA. Although it might be possible that the metabolism of CA 15-3 and CEA in pleural effusion and blood circulation differs from that in culture medium, it is likely that KPL-1 cells preferentially secrete TPA in an anchorage-dependent culture condition. Further experiments are needed to clarify and understand this phenomenon.

TPA is a protein antigen composed of more than 200 amino acids with a molecular weight of 17–43 kDa (Lüning *et al.*, 1980). This protein is suggested to be related to non-epidermal keratins 8, 18 and 19 (Weber *et al.*, 1984). Production and secretion of TPA from cultured HeLa cells has been reported to correlate with the early S-phase and the mitotic phase respectively (Björklund and Björklund, 1983). Thus, TPA is thought to be related to proliferative activity in general. TPA has been found not only in a variety of human malignancies, including breast cancer, but also in rapidly growing normal organs such as fetal tissues (Björklund and Björklund, 1983). Accumulated knowledge of TPA indicates that TPA may be a unique tumour-associated antigen in malignancies and also may be related to the regulatory mechanisms of cell growth. Preliminary results in the present study suggested that KPL-1 cells secrete a larger amount of TPA during the exponential growth phase than during the lag phase or the plateau phase. In contrast, the secretion of CA 15-3 seemed to be independent of any cell growth phase. Further analysis of KPL-1 cells, including the relationship between the cell cycle and secretion or production of tumour-associated antigens, should be done. Interestingly, the serum TPA concentration could be measured in the nude mice into which KPL-1 cells had been transplanted and the concentration linearly correlated with the volume of transplanted tumours (Figure 10). To the best of our knowledge, KPL-1 is the first cell line in which serum TPA has been detected *in vivo*. This cell line may make it possible to study the kinetics of tumour markers *in vivo* as well as *in vitro*.

It is known that breast cancer cell lines tend to be less tumorigenic and less metastatic than other cancer cell lines derived from lung, renal and colon carcinomas and sarcomas when the cell lines are subcutaneously injected into the flank of nude mice (Ozzello and Sordat, 1980; Shafie and Liotta, 1980; Price *et al.*, 1990). Recent reports suggest that orthotropic transplantation of cancer cell lines into nude mice is more tumorigenic and sometimes induces spontaneous metastasis (Fidler, 1991). In the present study, KPL-1 cells were injected orthotropically into the mammary fat pads of female nude mice. The tumour take rate of KPL-1 cells by this orthotropic transplantation was 100% and all the transplanted tumours grew rapidly (Figure 8). Moreover, micrometastases into the lymph nodes could be recognised in the nude mice with transplanted KPL-1 cells (Figure 9). These findings support the suggestion that orthotropic transplantation of cancer cell lines may be useful in creating tumours and spontaneous metastasis in nude mice.

Expression of ER is one of the most characteristic features of breast cancer. To clarify that KPL-1 cells are derived from breast cancer, ER in the transplanted KPL-1 tumours was measured by both the conventional ligand-binding assay and the enzyme immunoassay. A relatively large number of ERs were detected in the KPL-1 tumours by both methods. In contrast, no PRs were detected in the same tumours by these assays. ER-positive but PR-negative primary breast cancers are known to occur with a frequency of approximately 20% (McGuire and Horwitz, 1978). Recent reports suggest that aberration of a part of the ER gene, such as the DNA-binding domain, may produce tumours which express ER but not PR (Murphy and Dotzlaw, 1989; Wang and Miksicek, 1991; Fuqua *et al.*, 1992). Analysis of the ER gene of KPL-1 cells should be carried out to determine why the cells are ER positive but PR negative.

Another interesting point is that KPL-1 cells seem to grow oestrogen independently both *in vivo* and *in vitro*. The preliminary results in this study suggest that the anchorage-dependent growth of KPL-1 cells is not altered by the physiological concentration of $E_2$, a therapeutic concentration of an antioestrogen, TAM, or their combination (Figure 7). Further studies, such as an anchorage-independent growth experiment, are needed to clarify the hormone independency of KPL-1 cells *in vitro*. With regard to *in vivo* growth, it is well known that hormone-responsive MCF-7 cells are unable to grow well without oestradiol supplementation in intact female nude mice because of a low level of endogenous oestrogen (Soule and McGrath, 1980). This fact suggests that KPL-1 cells may grow oestrogen independently in female nude mice or that a low level of endogenous oestradiol may be sufficient to make KPL-1 cells grow in female nude mice. To address these questions, animal experiments using ovariectomised nude mice are underway.

Recently, some variants of the MCF-7 cell line have been reported to be ER positive but grow oestrogen independently in nude mice (Gottardis and Jordan, 1988; Clarke *et al.*, 1989; McLeskey *et al.*, 1993). One of these variants, the MKS-1 cell line, which overexpresses fibroblast growth factor 4, has been reported to show rapid growth in ovariectomised nude mice and maintain the expression of ER (McLeskey *et al.*, 1993). These findings suggest that activation of signal transduction via a certain growth factor or oncogene may influence the hormone dependency of breast cancer cell growth. To address this question, we investigated gene amplification of c-*erb*B-2, c-*myc*, H-*ras* and N-*ras* and expression of EGFR in KPL-1 cells. No amplification of any of the genes was detected in KPL-1 cells. Only a small amount of EGFR was detected. Further studies should be carried out to clarify the oestrogen independency of KPL-1 cells and elucidate its mechanisms.

In conclusion, we established a new human breast cancer cell line designated KPL-1 from the malignant effusion of a Japanese patient with recurrent breast cancer. Morphological, cytogenetic and biochemical analyses supported the conclusion that this cell line is derived from breast cancer. Preliminary characterisation of KPL-1 cells showed that this cell line possesses a large number of ERs but grows oestrogen independently *in vitro*. Furthermore, this cell line is highly tumorigenic in female athymic nude mice. Secretion of three tumour-assocaited antigens, CEA, CA 15-3 and TPA, was observed *in vivo* as well as *in vitro*. This cell line may be useful for studying not only the hormone independency of breast cancer but also the kinetics of tumour-associated antigens. Further characterisation of the KPL-1 cell line should be carried out in the near future.

**Acknowledgements**
The authors would like to thank Dr Robert B Dickson, Lombardi Cancer Research Center, Georgetown University, for his helpful comments on this manuscript as well as Mr Taiji Suda, Mrs Keiko Isoda and Mr Kenzo Uehara for technical assistance on the electron microscopic analysis. The animal protocol for these experiments was approved by the Animal Care and Use Committee of Kawasaki Medical School. This work was supported in part by a grant from the Ministry of Education, Science and Culture of Japan.

### References

ABE M AND KUFE D. (1987). Identification of a family of high molecular weight tumor associated glycoproteins. *J. Immunol.*, **139**, 257–261.

BJÖRKLUND B AND BJÖRKLUND V. (1983). Specificity and basis of the tissue polypeptide antigen. *Cancer Detect. Prev.*, **6**, 41–50.

CALLEAU R, YOUNG R, OLIVE M AND REEVES JR WJ. (1974). Breast tumor cell lines from pleural effusions. *J. Natl Cancer Inst.*, **53**, 661–674.

CHEN LC, KURISU W, LJUNG BM, GOLDMAN ES, MOORE D AND SMITH HS. (1992). Heterogeneity for allelic loss in human breast cancer. *J. Natl Cancer Inst.*, **84**, 506–510.

CLARKE R, BRÜNNER N, KAZTENELLENBOGEN BS, THOMPSON EW, NORMAN MJ, KOPPI C, PAIK S, LIPPMAN ME AND DICKSON RB. (1989). Progression of human breast cancer cells from hormone-dependent to hormone-independent growth *in vitro* and *in vivo*. *Proc. Natl Acad. Sci. USA*, **86**, 3649–3653.

ENGEL LW, YOUNG NA, TRALKA TS, LIPPMAN ME, O'BRIEN SJ AND JOYCE MJ. (1978). Establishment and characterization of three new continuous cell lines derived from human breast carcinomas. *Cancer Res.*, **38**, 3352–3364.

FIDLER IJ. (1991). 7th Jan Waldenström Lecture. The biology of human cancer metastasis. *Acta Oncol.*, **30**, 668–675.

FUQUA SAW, FITZGERALD SD, ALLRED DC, ELLEDGE RM, NAWAZ Z, McDONNELL DP, O'MALLEY BW, GREENE GL AND McGUIRE WL. (1992). Inhibition of estrogen receptor action by a naturally occurring variant in human breast tumors. *Cancer Res.*, **52**, 483–486.

GION M, MIONE R, GATTI C, DITTADI R, LEON A, NASCIMBEN O, PIZZORNO B AND BRUSCAGNIN G. (1990*a*). Is tissue polypeptide antigen still a useful tumor marker in breast carcinoma? Comparison with CA 15.3 and MCA. *Tumori*, **76**, 360–364.

GION M, MIONE R, GATTI C, DITTADI R, LEON A, CASTIGLIONI C, NASCIMBEN O AND BRUSCAGNIN G. (1990*b*). Tissue polypeptide antigen in tumor cytosol: a new prognostic indicator in primary breast cancer. *Breast Cancer Res. Treat.*, **17**, 15–21.

GOTTARDIS MM AND JORDAN VC. (1988). Development of tamoxifen-stimulated growth of MCF-7 tumors in athymic nude mice after long-term antiestrogen administration. *Cancer Res.*, **48**, 5183–5187.

HAYES DF, ZURAWSKI VR AND KUFE DW. (1986). Comparison of circulating Ca 15-3 and carcinomembryonic antigen in patients with breast cancer. *J. Clin. Oncol.*, **4**, 1542–1550.

HORWITZ KB. (1992). Cellular heterogeneity and mutant oestrogen receptors in hormone resistant breast cancer. *Cancer Surv.*, **14**, 41–54.

KINOSHITA M, KATSURAGI K, SHIN S AND AONO T. (1994). Amplification and point-mutation alterations of oncogenes in patients with cervical cancer-associated human papillomavirus. *Biomed. Res.*, **14**, 403–410.

KUFE D, INGHIRAMI G, ABE M, HAYES D, JUSTI-WHEEKER H AND SCHLOM J. (1984). Differential reactivity of a novel monoclonal antibody (DF3) with human malignant versus benign breast tumors. *Hybridoma*, **3**, 223–232.

KUREBAYASHI J, ISHIDA T, HIGASHI Y, SUEMASU K, NOMOTO C AND YOSHIDA K. (1990). Prognostic significance of estrogen and progesterone receptors in primary operable breast cancer. *Jpn J. Clin. Oncol.*, **20**, 271–280.

LÜNING B, WIKLUND B, REDELIUS P AND BJÖRKLUND B. (1980). Biochemical properties of tissue polypeptide antigen. *Biochim. Biophys. Acta*, **624**, 90–101.

McGUIRE WL AND HORWITZ KB. (1978). Progesterone receptors in breast cancer. In *Hormones, Receptors, and Breast Cancer*, McGuire WL (ed.) pp. 31–42. Raven Press: New York.

McLESKEY SW, KUREBAYASHI J, HONIG SF, ZWIEBEL J, LIPPMAN ME, DICKSON RB AND KERN FG. (1993). Fibroblast growth factor 4 transfection of MCF-7 cells produces cell lines that are tumorigenic and metastatic in ovariectomized or tamoxifen-treated athymic nude mice. *Cancer Res.*, **53**, 2168–2177.

MROSS K AND BANDLOW G. (1986). The clinical significance of tissue polypeptide antigen in breast cancer patients. *Anticancer Res.*, **6**, 223–226.

MURPHY LC AND DOTZLAW H. (1989). Variant estrogen receptor mRNA species detected in human breast cancer biopsy samples. *Mol. Endocrinol.*, **3**, 687–693.

NEMOTO T, CONSTANTINE R AND CHU TM. (1979). Human tissue polypeptide antigen in breast cancer. *J. Natl Cancer Inst.*, **63**, 1347–1350.

OZZELLO L AND SORDAT M. (1980). Behavior of tumors produced by transplantation of human mammary cell lines in athymic nude mice. *Eur. J. Cancer*, **16**, 553–559.

PRICE JE, POLYZOS A, ZHANG RD AND DANIELS LM. (1990). Tumorigenicity and metastasis of human breast carcinoma cell lines in nude mice. *Cancer Res.*, **50**, 717–721.

SHAFIE SM AND LIOTTA LA. (1980). Formation of metastasis by human breast carcinoma cells (MCF-7) in nude mice. *Cancer Lett.*, **11**, 81–87.

SOULE HD, VAZQUEZ J, LONG A, ALBERT S AND BRENNAN M. (1973). A human cell line from a pleural effusion derived from a breast carcinoma. *J. Natl Cancer Inst.*, **51**, 1409–1416.

SOULE HD AND McGRATH CM. (1980). Estrogen responsive proliferation of clonal human breast carcinoma cells in athymic mice. *Cancer Lett.*, **10**, 177–189.

SCHOLL SM, HUFF KK AND LIPPMAN ME. (1983). Antiestrogenic effects of LY117018 in MCF-7 cells. *Endocrinology*, **113**, 611–617.

WANG Y AND MIKSICEK RJ. (1991). Identification of a dominant negative form of the human estrogen receptor. *Mol. Endocrinol.*, **5**, 1707–1715.

WEBER K, OSBORN M, MOLL R, WIKLUNK B AND LÜNING B. (1984). Tissue polypeptide antigen (TPA) is related to the non-epidermal keratins 8, 18 and 19 typical of simple and non-squamous epithelia: re-evaluation of a human tumor marker. *EMBO J.*, **3**, 2707–2714.

WOLMAN SR AND DAWSON PJ. (1991). Genetic events in breast cancer and their clinical correlates. *Crit. Rev. Oncol.*, **2**, 277–291.

YASUI W, SUMIYOSHI H, HATA J, KAMEDA T, OCHIAI A, ITO H AND TAHARA E. (1988). Expression of epidermal growth factor receptor in human gastric and colonic carcinomas. *Cancer Res.*, **48**, 137–141.